# Reclamation of a lignite combustion waste disposal site with alders (*Alnus* sp.): assessment of tree growth and nutrient status within 10 years of the experiment

**DOI:** 10.1007/s11356-018-1892-7

**Published:** 2018-04-11

**Authors:** Marcin Pietrzykowski, Bartłomiej Woś, Marek Pająk, Tomasz Wanic, Wojciech Krzaklewski, Marcin Chodak

**Affiliations:** 1 Department of Forest Ecology and Reclamation, Institute of Ecology and Silviculture, Faculty of Forestry, University of Agriculture in Krakow, Al. 29 Listopada 46, 31–425, Krakow, Poland; 2 Department of Soil Science, Institute of Ecology and Silviculture, Faculty of Forestry, University of Agriculture in Krakow, Al. 29 Listopada 46, 31–425, Krakow, Poland; 3 Department of Environmental Management and Protection, AGH University of Science and Technology, al. Mickiewicza 30, 30–059, Krakow, Poland

**Keywords:** Fly ash, Lignite, Alders, Revegetation

## Abstract

Combustion wastes are characterised by extremely low N contents. Therefore, introduction of nitrogen-fixing species at the first stage of their biological reclamation is required. This paper presents an assessment of the growth parameters of alders (*Alnus* sp.) 10 years after their introduction to a disposal site of lignite combustion waste in Central Poland. Black (*Alnus glutinosa*) and grey alders (*Alnus incana*) were planted directly in the combustion waste. The soil amendment included three variants: control with pure combustion waste, admixture of lignite culm and addition of acid sand. Both alder species displayed good growth parameters comparable to those of alders in natural habitats. However, black alder had better growth parameters, such as stand density index (SDI), diameter at breast height (DBH) and height (H) than grey alder. The lignite amendment exerted a positive effect on tree growth, reflected in a higher SDI and H, whereas the acid sand amendment did not affect any of the growth parameters of the studied alder species. Despite the good growth parameters, the measured N:P and N:K ratios in the alder leaves largely differed from the optimal values indicating insufficient P and K supply at the combustion waste disposal site. This may pose a threat to further development of the introduced tree plantings. The introduction of alders along with the lignite addition into the planting holes seems to be a successful method of combustion waste revegetation.

## Introduction

Combustion waste disposal sites may adversely affect the environment in adjacent areas because of wind erosion and dust pollution as well as leaching of alkaline and saline water which may lead to ground water contamination (Ram et al. 2008; Cho and Park 2018). Ash from landfills remains suspended in the air for a long time and may negatively affect the health of the local population (Dellantonio et al. 2009; Pandey et al. 2009), and dust from ash disposal sites negatively affects the adjacent ecosystems, mainly through soil alkalisation and changes in soil morphology and properties (Frąc et al. 2017; Weber et al. 2017).

Revegetation of combustion waste disposal sites by planting shrubs and trees on their slopes and tops could be an effective way to limit their adverse influences on the environment (Pandey 2015; Żołnierz et al. 2016). However, introduction of vegetation onto the combustion waste is difficult due to the negative physicochemical properties of these materials, such as high susceptibility to compaction, poor air and water ratios, excessively alkaline reaction, high EC variability, lack of nitrogen and available phosphorus and in some cases, high content of heavy metals (Haynes 2009; Weber et al. 2015). In Central Europe, several tree species have been introduced onto combustion waste disposal sites with various results (Pietrzykowski et al. 2010; Żołnierz et al. 2016). However, information on the growth of these introduced trees over a long time period and the long-term effects of planted trees on the properties of combustion wastes is still missing (Krzaklewski et al. 2012; Pietrzykowski et al. 2015). There are some studies on the reclamation of combustion wastes; however, most of these studies have been carried out on ashes covered with a layer of mineral soil (Čermák, 2008; Pietrzykowski et al. 2010). This practice is expensive and entails the risk of root system deformation due to the fact that roots develop primarily in the surface horizons containing mineral soil (Čermák, 2008). For instance, Čermák (2008) observed a significant deformation of root systems of Scots pine and black alder when a 0.4–0.5-m layer of mineral soil was used to cover ash deposits. As root deformation may adversely affect the stability of introduced tree stands in later phases of development, a possible method for tree introduction directly into the ash is still needed (Krzaklewski et al. 2012).

Alders (*Alnus* sp.) are promising tree species that could be used in biological restoration of combustion waste disposal sites (Krzaklewski et al. 2012; Pietrzykowski et al. 2015). These pioneering species often occur on former agricultural lands under secondary succession (Vacek et al. 2016). The symbiosis with nodule-forming bacteria capable of atmospheric N fixation makes them valuable in the reclamation of post-mining sites (Krzaklewski et al. 2012). Owing to their intensive litterfall and rooting alders increase the amount of soil organic matter (SOM) and the bioavailability of nutrients, acting as a natural “fertiliser” in former industrial sites (Sroka et al. 2017).

Different alder species have been used in the reclamation of former industrial sites in a wide range of conditions and soil substrates (Józefowska et al. 2016; Plamping et al. 2017). Čermák (2008) reported that black alder planted in combustion waste sedimentation ponds covered with a 0.4–0.5-m layer of mineral soil had good growth parameters. However, trials of the introduction of black and grey alders directly onto a sedimentation pond for ashes from bituminous coal combustion in Halemba power plant (southern Poland) and in a lignite power plant in Konin (central Poland) carried out in 1970s were less successful and indicated low survival rates and poor growth of the introduced alder species (Wysocki 1979). Only recently, Krzaklewski et al. (2012) suggested that black and grey alders can be successfully used in the biological restoration of lignite combustion waste disposal sites, and Pietrzykowski et al. (2015) pointed out that green alder (*Alnus viridis*) may also be a prospective species for biological reclamation and anti-erosion protection. Large-scale application of alder species for reclamation of ash deposition sites requires detailed knowledge on the survival and growth rate and size of the biomass of different alder species. Furthermore, determination of the optimal time for the growth of alders as a forecrop is needed to replace them with target species.

In this study, we assessed the adaptation of alders to a combustion disposal site within 10 years of the introduction based on their growth parameters and foliage nutrient status. Growth parameters were compared with a preliminary assessment carried out 5 years previously during the initial period of tree development (Krzaklewski et al. 2012).

## Materials and methods

### Study site

Lubień combustion waste disposal site is located in Central Poland (N 51° 27′, E 19° 27′), in a temperate climate zone with precipitation ranging from 550 to 600 mm annually and an average annual temperature of around 7.6–8.0 °C. The vegetation period lasts from 210 to 218 days (Woś 1999). The Lubień disposal site has been in operation since 1980 and currently occupies ca. 440 ha. Waste (ashes) from lignite combustion is transported by a hydro-transport installation to the surface of the site in an aqueous suspension, and there, it is deposited in segments restricted by dykes. After draining and evaporation of excess water, the waste accumulates higher and higher. Combustion waste containing about 85% ash and 15% slag is deposited by hydro-transport. The main components of combustion waste are thermally processed aluminosilicates. The average content of Al_2_O_3_ and SiO_2_ compounds ranges from about 60 to 70%, and that of calcium oxide CaO is about 20%. The content of trace elements (Zn, Cu, Pb, Cd, Cr) generally does not exceed the average reported for natural soils (Krzaklewski et al. 2012). In the case of the Lubień disposal site, adverse environmental impact is caused mainly by leaching of sulphate, chloride and calcium, which in turn increases the concentrations of these ions, and overall hardness and alkalinisation of ground water (Krzaklewski et al. 2012).

### Description of the study

The study commenced in September 2005 in a part of the combustion waste disposal site flat shelf set up between 2003 and 2004. Prior to the planting of trees, the study site was subjected to hydro-seeding with sewage sludge (4 Mg dry mass ha^−1^) mixed with the seeds (200 kg ha^−1^) of Cock’s foot grass (*Dactylis glomerata* L.) and Italian ryegrass (*Lolium multiflorum* Lam.). Next, NPK start-up mineral fertilisation was applied with N 60 kg ha^−1^, P 36 kg ha^−1^ and K 36 kg ha^−1^. Subsequently, 24 plots (6 × 13 m each), separated by a 2-m-wide buffer strip (not planted and covered by grasses), were laid out. Within each plot, 50 seedlings of black or grey alder (6410 trees ha^−1^) were planted into 40 × 40 × 40-cm holes in three treatment variants (with four replications for each variant): with addition (3 dm^3^ in planting hole) of lignite (CCW + L), Miocene acid sand admixture (CCW + MS) and control without any soil amendment (CCW) (Krzaklewski et al. 2012). Lignite and acid sand were added to support the adaptation and growth of seedlings in the initial period of growth on the alkaline combustion waste. Lignite was used as an amendment owing to its high content of humic substances and nutrients (Kwiatkowska et al. 2008). Acid sand was added to reduce the extremely high pH of the combustion waste. The applied amendments were chosen as they were available in the direct vicinity of the combustion waste disposal site. Power stations are usually located close to lignite mines, and tertiary acid sands are found in the overburden of lignite deposits.

### Soil sampling and analyses

Soil samples were collected in 2015 from 0- to 40-cm horizon at five points within each study plot; four of them were located in the corners and one in the centre of the plot. The samples were bulked to produce a mixed sample (1.0-kg mass of fresh materials) representative for the sampling plot. Twenty-four mixed samples were used to determine basic soil properties.

The soil samples were dried and sieved through a 2.0-mm sieve and measured for texture with a Fritsch GmbH Laser Particle Sizer ANALYSETTE 22, pH in 1 M KCl (1:2.5 soil:solution ratio) and electrical conductivity (EC) (1:5 soil:solution ratio at 21 °C). Organic carbon (OC), total nitrogen (Nt) and sulphur (St) content were determined with LECO TruMac® CNS analyser. Prior to carbon analysis, the soil samples were treated with 10% HCl to remove carbonates. Exchangeable acidity (Hh) was determined in 1 M Ca(OAc)_2_ and basic exchangeable cations (Ca^2+^, Mg^2+^, K^+^, Na^+^) in 1 M NH_4_Ac by ICP-OES (iCAP^™^ 6000 Series); available phosphorus (Pav) was assayed in calcium lactate extract ((CH_3_CHOHCOO)_2_Ca) acidified with HCl to pH 3.6 (Egner-Riehm method) by Varian CARY 300 Conc UV-Visible spectrophotometer. CEC was determined by the sum of exchangeable cations and Hh. The total contents of Mn, Zn, Cu, Pb, Cd, Ni and Cr were determined after digestion in a mixture of HNO_3_ (*d* = 1.40) and 60% HClO_4_ acid in a 4:1 proportion, by ICP-OES (iCAP^™^ 6000 Series).

### Tree foliage sampling and analyses

Leaves were collected in summer from five trees regularly distributed along the diagonal of each plot from crown tops of the SW exposition (Baule and Fricker 1970). The collected leaves from each plot were mixed to produce a mixed sample representative for the plot.

The contents of C, N and S in the leaves were measured with the LECO TruMac® CNS analyser. Total Ca, Mg, K, P and trace elements (Zn, Cu, Cd, Pb) in the leaves were determined by atomic absorption spectroscopy ICP-OES (iCAP^™^ 6000 Series) after digestion in a mixture of HNO_3_ (*d* = 1.40) and 60% HClO_4_ acid in a 4:1 ratio (Ostrowska et al. 1991).

### Assessment of tree growth parameters

In each study plot, the survival of trees as stand density (*D* − number of trees per ha) was assessed, and diameter at breast height (DBH) with an accuracy of 0.1 cm and height (*H*) of all trees with an accuracy of 0.01 m were measured. Tree measurements were conducted in autumn 2015 after 10 years of growth. Based on these measurements and results from previous measurements in spring 2011 (Krzaklewski et al. 2012), the current annual growth was calculated for tree height (Δ*H*) and change of stand density (Δ*D*) from spring 2011 to autumn 2015. In addition, the upper height (H100) was determined, i.e. the average height of the 100 tallest trees per hectare. The Reineke’s stand density index (SDI) determined from the equation (Zeide 2005):

$$ \mathrm{SDI}=D\bullet {\left(\frac{\mathrm{DBH}}{25}\right)}^{1.6} $$

where SDI is the stand density index, *D* is the density (number of trees per hectare) and DBH is the diameter at breast height.

### Statistical analyses

Two-way analysis of variance (ANOVA) was used to test the effect of the soil treatment and tree species on alder growth parameters (*D*, Δ*D*, SDI, DBH, *H*, Δ*H* and H100). Prior to ANOVA, the data sets were tested for normality using the Kolmogorov-Smirnov test and for variance homogeneity using Leven’s test. Tukey’s honestly significant difference (HSD) test for unequal sample sizes was run if any significant differences were found (*p* < 0.05). We used STATISTICA version 13.1 software (StatSoft Inc. 2017).

## Results

### Soil characteristics

Studied technosols displayed low silt (7–8%) and clay (1%) contents, neutral or slightly alkaline reaction (pH 7.3–7.4) and low EC (185–206 μS cm^−1^). The OC content ranged from 3.14 to 4.73%, the Nt content from 0.029 to 0.043%, the St content from 0.067 to 0.076% and the Pav content from 10.08 to 11.13 mg kg^−1^. The CEC was from 35.71 to 39.23 cmol(+) kg^−1^, with Ca being the most abundant cation (34.13 to 37.52 cmol(+) kg^−1^). The BS was high ranging from 97.39 to 98.14% (Table 1).

**Table 1:** Effects of treatment (CCW, CCW + L, CCW + MS) and tree species on soil properties by two-way ANOVA

Factor	Level	Silt	Clay	CaCO_3_	pH_KCl_	EC	Nt	OC	St	Pav	Ca^2+^	Mg^2=^	K^+^	Na^2=^	Hh	CEC	BS
						μS cm^−1^	%			mg kg^−1^	cmol(+) kg^−1^						%
		%															
		N.S.*	N.S.	N.S.	N.S.	N.S.	N.S.	N.S.	N.S.	N.S.	N.S.	N.S.	N.S.	N.S.	N.S.	N.S.	N.S.
Tree species (Sp)	Black alder	8 ± 0.9^a^	1 ± 0.1	3.36 ± 0.41	7.4 ± 0.04	185 ± 15	0.032 ± 0.005	3.37 ± 0.48	0.069 ± 0.008	10.51 ± 1.12	37.52 ± 2.46	0.77 ± 0.05	0.09 ± 0.01	0.08 ± 0.01	0.77 ± 0.04	39.23 ± 2.51	97.83 ± 0.24
	Grey alder	7 ± 0.9	1 ± 0.1	3.24 ± 0.39	7.3 ± 0.03	196 ± 14	0.044 ± 0.005	3.91 ± 0.45	0.074 ± 0.008	10.12 ± 1.06	34.13 ± 2.46	0.71 ± 0.07	0.07 ± 0.01	0.08 ± 0.01	0.73 ± 0.04	35.71 ± 2.54	97.76 ± 0.19
		N.S.	N.S.	N.S.	N.S.	N.S.	N.S.	N.S.	N.S.	N.S.	N.S.	N.S.	N.S.	N.S.	N.S.	N.S.	N.S.
Soil treatment (Tr)	CCW	7 ± 1.0	1 ± 0.2	2.83 ± 0.49	7.4 ± 0.04	186 ± 17	0.029 ± 0.006	3.14 ± 0.56	0.067 ± 0.010	10.63 ± 1.24	37.13 ± 3.20	0.78 ± 0.08	0.07 ± 0.01	0.09 ± 0.01	0.76 ± 0.06	38.83 ± 3.28	97.86 ± 0.26
	CCW + L	8 ± 1.0	1 ± 0.2	4.15 ± 0.49	7.3 ± 0.04	178 ± 17	0.043 ± 0.006	4.73 ± 0.56	0.072 ± 0.010	10.08 ± 1.31	36.18 ± 2.38	0.72 ± 0.07	0.07 ± 0.01	0.07 ± 0.01	0.66 ± 0.02	37.7 ± 2.46	98.14 ± 0.14
	CCW + MS	8 ± 1.1	1 ± 0.2	2.93 ± 0.41	7.4 ± 0.05	206 ± 18	0.041 ± 0.006	3.74 ± 0.60	0.076 ± 0.011	11.13 ± 2.16	34.17 ± 3.63	0.72 ± 0.09	0.09 ± 0.01	0.07 ± 0.01	0.83 ± 0.04	35.87 ± 3.74	97.39 ± 0.30
Interaction	Tr × Sp	N.S.	N.S.	N.S.	N.S.	N.S.	N.S.	N.S.	N.S.	N.S.	N.S.	N.S.	N.S.	N.S.	N.S.	N.S.	N.S.

*p < 0.05, *N.S.* differences not significant

^a^Mean ± SE

The total contents of trace elements were relatively low: Mn 93.95–112.70 mg kg^−1^, Zn 34.26–40.64 mg kg^−1^, Cu 12.97–17.12 mg kg^−1^, Cd 0.79–0.90 mg kg^−1^, Pb 32.51–40.00 mg kg^−1^, Cr 14.94–19.59 mg kg^−1^ and Ni 18.82–22.17 mg kg^−1^ (Table 2).

**Table 2:** Effects of treatment (CCW, CCW + L, CCW + MS) and tree species on trace element concentration in soil (0–40 cm) by two-way ANOVA

Factor	Level	Mn	Zn	Cu	Cd	Pb	Cr	Ni
		mg kg^−1^						
		N.S.*	N.S.	N.S.	N.S.	N.S.	N.S.	N.S.
Tree species (Sp)	Black alder	106.45 ± 8.35^a^	37.22 ± 2.50	15.39 ± 1.75	0.89 ± 0.07	38.57 ± 3.12	15.86 ± 0.59	19.00 ± 0.91
	Grey alder	103.48 ± 6.33	36.33 ± 2.75	14.04 ± 2.24	0.84 ± 0.07	32.49 ± 1.72	17.28 ± 2.47	21.10 ± 2.19
		N.S.	N.S.	N.S.	N.S.	N.S.	N.S.	N.S.
Soil treatment (Tr)	CCW	108.25 ± 8.87	34.26 ± 3.44	17.12 ± 1.89	0.90 ± 0.11	34.08 ± 2.64	14.94 ± 0.51	18.82 ± 1.29
	CCW + L	112.70 ± 6.88	35.42 ± 2.71	14.05 ± 2.74	0.90 ± 0.07	40.00 ± 4.01	19.59 ± 3.57	22.17 ± 2.97
	CCW + MS	93.95 ± 10.29	40.64 ± 3.17	12.97 ± 2.61	0.79 ± 0.09	32.51 ± 2.54	15.18 ± 0.68	19.16 ± 1.52
Interaction	Tr × Sp	N.S.	N.S.	N.S.	N.S.	N.S.	N.S.	N.S.

*p < 0.05, *N.S.* differences not significant

^a^Mean ± SE

There were no differences after 10 years in the chemical properties of soil under different tree species or soil treatments.

### Tree growth parameters

After 10 years of growth, black alder displayed better growth parameters compared with grey alder: *D* was 4423 and 4583 trees ha^−1^, and Δ*D* in the period 2011–2015 was − 81 and − 141 trees ha^−1^ yr^−1^, respectively, for black and grey alder. The SDI was 507.6 and 234.5, DBH 6.4 and 3.9 cm, *H* 6.3 and 3.9 m, Δ*H* in the period from 2011 to 2015 was 0.65 and 0.29 m yr^−1^ and H100 9.0 and 8.5 m, respectively, for black and grey alder. Differences were statistically significant (*p* < 0.05) for SDI, DBH, *H* and Δ*H*.

There were no significant interactions between the tree species and the type of soil substrate (Tr × Sp) (Table 2). A significantly higher SDI index was found in CCW + L (424.5) compared with CCW (321.3). The *H* was also higher in CCW + L (5.6 m) compared with CFA (4.9 m) and CCW + MS (5.0 m) (Table 3).

**Table 3:** Effects of treatment (CCW, CCW + L, CCW + MS) and tree species on alder growth parameters on the combustion waste landfill by two-way ANOVA

Factor	Level	*D*	Δ*D*^b^	SDI	DBH	*H*	Δ*H*^b^	H100
		trees ha^−1^	trees ha^−1^ yr^−1^		cm	m	m yr^−1^	m
		N.S.	N.S.	*	*	*	*	N.S.
Tree species (Sp)	Black alder	4423±714^a^	− 81 ± 102	507.6 ± 109.8 b	6.4 ± 0.2 b	6.3 ± 0.2 b	0.65 ± 0.11 b	9.0 ± 1.0
	Grey alder	4583 ± 798	− 141 ± 80	234.5 ± 50.9 a	3.9 ± 0.2 a	3.9 ± 0.2 a	0.29 ± 0.11 a	8.5 ± 1.3
		N.S.	N.S.	*	N.S.	*	N.S.	N.S.
Soil treatment (Tr)	CCW	4407 ± 699	− 147 ± 110	321.3 ± 157.6 a	4.8 ± 0.2	4.9 ± 0.3 a	0.43 ± 0.20	8.2 ± 1.0
	CCW + L	4888 ± 835	− 112 ± 63	424.5 ± 179.01 b	5.4 ± 0.2	5.6 ± 0.2 b	0.51 ± 0.21	9.1 ± 1.4
	CCW + MS	4214 ± 603	− 73 ± 101	367.4 ± 154.8 ab	5.4 ± 0.2	5.0 ± 0.2 a	0.46 ± 0.25	9.0 ± 1.2
Interaction	Tr × Sp	N.S.	N.S.	N.S.	N.S.	N.S.	N.S.	N.S.

Different letters indicate significant (*p* < 0.05) differences between the studied variants

*p < 0.05, *N.S.* differences not significant

^a^Mean ± SE

^b^ΔN and ΔH calculated as differences between values in 2011 and 2015

### Tree foliage element content

The macronutrient content in the alder leaves was similar between the studied species and amounted to N 2.81 and 2.49%, P 0.14 and 0.13%, K 0.39 and 0.36%, Ca 2.16 and 2.14%, Mg 0.31 and 0.32%, Na 0.004 and 0.003% and S 0.25 and 0.24% for black and grey alder, respectively. There was no significant effect of soil treatment on alder nutrient supply (Table 4).

**Table 4:** Effects of treatment (CCW, CCW + L, CCW + MS) and tree species on element content in foliage of alders on combustion waste landfill by two-way ANOVA

Factor	Level	N	P	K	Ca	Mg	Na	S	Mn	Zn	Cu	Cd	Pb	Cr	Ni
		%							mg kg^−1^						
		N.S.	N.S.	N.S.	N.S.	N.S.	N.S.	N.S.	*	*	*	N.S.	*	N.S.	*
Tree species (Sp)	Black alder	2.81 ± 0.07^a^	0.14 ± 0.01	0.39 ± 0.01	2.16 ± 0.05	0.31 ± 0.02	0.004 ± 0.001	0.25 ± 0.01	24.08 ± 1.66 a	46.31 ± 1.88 a	5.19 ± 0.32 a	0.14 ± 0.01	3.82 ± 0.18 b	4.45 ± 0.57	2.86 ± 0.19 a
	Grey alder	2.49 ± 0.06	0.13 ± 0.01	0.36 ± 0.02	2.14 ± 0.09	0.32 ± 0.02	0.003 ± 0.001	0.24 ± 0.01	35.44 ± 2.28 b	61.29 ± 3.27 b	7.23 ± 0.49 b	0.12 ± 0.01	3.09 ± 0.15 a	3.50 ± 0.24	3.37 ± 0.13 b
		N.S.	N.S.	N.S.	N.S.	N.S.	N.S.	N.S.	N.S.	N.S.	N.S.	N.S.	N.S.	N.S.	N.S.
Soil treatment (Tr)	CCW	2.64 ± 0.11	0.13 ± 0.01	0.38 ± 0.02	2.09 ± 0.06	0.33 ± 0.02	0.004 ± 0.001	0.24 ± 0.01	29.30 ± 3.21	56.05 ± 5.04	6.27 ± 0.57	0.13 ± 0.01	3.30 ± 0.28	4.31 ± 0.68	3.12 ± 0.29
	CCW + L	2.67 ± 0.09	0.13 ± 0.01	0.38 ± 0.02	2.30 ± 0.11	0.31 ± 0.02	0.004 ± 0.001	0.24 ± 0.01	32.48 ± 3.56	50.95 ± 4.18	6.75 ± 0.69	0.14 ± 0.01	3.68 ± 0.22	4.61 ± 0.44	3.08 ± 0.22
	CCW + MS	2.64 ± 0.1	0.14 ± 0.01	0.37 ± 0.02	2.07 ± 0.07	0.30 ± 0.02	0.003 ± 0.001	0.24 ± 0.01	27.49 ± 2.78	54.41 ± 3.56	5.60 ± 0.62	0.12 ± 0.01	3.39 ± 0.22	3.01 ± 0.36	3.15 ± 0.14
Interaction	Tr × Sp	N.S.	N.S.	N.S.	N.S.	N.S.	N.S.	N.S.	N.S.	N.S.	N.S.	N.S.	N.S.	N.S.	N.S.

Different letters indicate significant (*p* < 0.05) differences between the studied variants

*p < 0.05, *N.S.* differences not significant

^a^Mean ± SE

Significantly higher average concentrations of trace elements in grey alder leaves compared with black alder were found for Mn, Zn, Cu and Ni, whilst for Pb, the opposite was the case (Table 4). However, there was no effect observed of the soil treatment on the concentration of trace elements (Table 4).

## Discussion

The growth parameters of tree species are associated with their adaptation to the habitat conditions of anthropogenically transformed sites (Kuznetsova et al. 2011; Pietrzykowski and Socha 2011). At 10 years after planting, black alder displayed better growth parameters than grey alder. Poorer growth parameters of grey alder may be due to its ecological properties. In Central European conditions, grey alder is smaller than black alder (Boratyński 1980). However, when compared to their size in natural habitats, both species displayed good growth parameters indicating their good adaptability. In the optimal forest habitats in southern Poland, black alder aged from 5 to 9 years had *D* 3900–5200 trees ha^−1^, DBH 2.7–4.9 cm and HL 4.0–8.0 m (Orzeł et al. 2005). Grey alder stands in the Baltic states (Estonia) at the age of 10–12 years had *D* 6266–15,900 trees ha^−1^, DBH 3.7–4.6 cm and *H* 5.9–8.3 m (Uri et al. 2014). However, at combustion waste disposal sites, maximising the growth of introduced alder species is not the most important goal. The main role of alders is to reduce erosion of disposal sites and to transform the combustion wastes into soil (Krzaklewski et al. 2012). The latter function is connected with the ability of alders to produce large amounts of easily degradable, nutrient-rich organic residues resulting in a faster rate of soil organic matter and N accumulation (Sroka et al. 2017). In the harsh habitat conditions of combustion waste disposal sites, a dying out of alders, similar to that on reclaimed mining sites, may occur in the future. Observations from afforested post-mining sites where alders were planted as a soil ameliorative admixture indicated their gradual regression and dying out within approximately 15–20 years. This process was due to both the habitat conditions, which were far from alders ecological optimum (rainfall-retention water management) and from the competition with target tree species (Pietrzykowski 2015). Nevertheless, during 15–20 years of growth, planted alders play an important role in shaping the biotope and preparing it for the target species (Pietrzykowski 2015).

The applied soil amendment variants used had limited effect on the growth parameters of black and grey alders indicating that mineral fertilisation with NPK at the onset followed by hydro-seeding with sewage sludge and a mixture of grass seeds is sufficient to initiate the growth of alder plantings. However, the higher SDI index and *H* in the CCW + L variant suggest that a lignite admixture should be applied for the best results. Tree height is widely used as an indicator of habitat fertility (Socha 2008), and the SDI index values express density of a stand in terms of an equivalent number of trees with a 24.5-cm diameter (Zeide 2005). In an earlier study, Krzaklewski et al. (2012) after 5 years of alders’ growth also highlighted that the best way to improve the properties of ashes for alder growth and biomass production was to add lignite to the planting holes.

The acid sand admixture did not affect any of the analysed growth parameters. This confirms earlier observations that the applied sand admixture was not enough to decrease the strongly alkaline pH of the combustion waste and improve the properties of combustion waste for alder growth (Krzaklewski et al. 2012). However, black and grey alders have been successfully introduced on alkaline post-mining deposits such as tertiary clays of sulphur mine overburden (Józefowska et al. 2016) and deposits accompanying oil shale opencast mines (Kuznetsova et al. 2011). Apparently, the alkaline soil reaction does not adversely affect growth of the analysed alder species.

An earlier study on the introduction of grey and black alders on disposal sites containing bituminous coal and lignite combustion did not bring positive results due to the low survival rate and poor tree growth after 3 years (Wysocki 1979). However, site preparation for planting was different than in our study and included various fertilisation combinations: admixture of fertile soil, tertiary acid sands, bentonite and peat. To protect from erosion, the site was sprayed with latex emulsions and a mix of herbaceous plants was sown. Failure of that trial might have resulted also from severe weather conditions (drought and low temperatures) and the impact of industrial pollution in 70, in particular by sulphur compounds and the resulting acid rain (Wysocki 1979).

Combustion wastes are often characterised by extremely low N contents, which may lead to insufficient N supply for trees (Rai et al. 2004). Therefore, measurement of the N content in leaves is important to assess the adaptation of tree species to infertile technosols on post-industrial sites (Kuznetsova et al. 2011; Pietrzykowski et al. 2013). The measured nitrogen content in the leaves of black and grey alders in this study was in the range (2.6% for grey alder and 2.9% for black alder) previously reported for this site (Krzaklewski et al. 2012). The measured N contents in the alder leaves were similar to contents measured for alders growing on former agricultural sites in Central Europe (Uri et al. 2002) and nurseries in western Poland (Lorenc-Plucińska et al. 2013) and only slightly lower than those reported for the leaves of 7-year-old black alders growing on the reclaimed areas of oil shale opencast mines in Northeast Estonia (Kuznetsova et al. 2011). This indicates that N supply at the studied combustion waste pond is sufficient for planted grey and black alders.

The contents of P and K in the leaves of grey and black alders were in the range typically reported for alders growing on different natural and reclaimed soils (Kuznetsova et al. 2011; Lorenc-Plucińska et al. 2013). However, both the contents of particular elements in foliage and their ratios are important for the assessment of nutrient supply to trees (Baule and Fricker 1970; Ingestad 1987). Balanced nutrient supply means that each component should occur in an appropriate proportion to other nutrients (Marschner 2012). According to Ingestad (1987), in grey alder leaves, the ratio of basic macronutrients, N:P:K:Ca:Mg, required for optimal nutrition and maximum growth is 100:18:50:5:9. In our study, this ratio strongly deviated from the optimal values and was 100:5:14:77:11 for black alder and 100:5:15:76:11 for grey alder. The low concentrations of P and K in the alder leaves might have resulted from the low contents of these elements in the combustion wastes. However, high pH and Ca concentration might have additionally contributed to the insufficient uptake of these elements. At high pH and Ca, content availability of P is low as phosphate ions react rapidly with Ca ions to form insoluble compounds (Hinsinger 2001). High Ca may also negatively affect the uptake of K probably owing to decreased permeability of cells (Fageria 2001).

The studied combustion waste did not contain trace elements at concentrations regarded toxic to plants (Kabata-Pendias 2011). The concentrations of trace elements in the leaves of analysed alder species also did not exceed the amounts considered toxic (Kabata-Pendias 2011). Further, there was no deficiency of trace elements with an important physiological role in plants (Mn, Zn, Cu and Ni) (Marschner 2012; Kabata-Pendias 2011), wherein grey alder displayed significantly higher concentrations of Mn, Zn, Cu and Ni in the leaves compared with black alder.

## Conclusions

The study indicated good growth parameters of black and grey alders planted on a combustion waste disposal site. After 10 years of growth, the introduced species exhibited major growth parameters (density, SDI index, average diameter at breast height and height and increase in height) comparable with alders of similar age growing in natural habitats. Black alder had better growth parameters than grey alder, probably due to different ecological characteristics and growth rate during the early periods of growth. The acid sand amendment did not affect growth parameters of the studied alder species but the lignite amendment resulted in higher SDI and tree height. Therefore, initial NPK mineral fertilisation, hydro-seeding with sewage sludge along with an admixture of lignite to the planting holes is recommended to improve the growth of alders on reclaimed combustion waste sites. Despite good growth, the chemical analyses indicated disturbed proportions of some elements in the foliage. In particular, shortage of phosphorus and potassium may pose a threat to the stability of the investigated tree species in the later periods of growth.
